# A Study of Leprosy

**Published:** 1884-08

**Authors:** 


### A STUDY OF LEPROSY.

DR. G. H. Fox, the lecturer on cutaneous diseases at the College of Physicians and Surgeons, New York City, has recently made a trip to a leprosy lazaretto at Tracadie, Province of New Brunswick. In an interview with a representative of the New York *Sun* Dr. Fox gives some interesting information about this disease, which is less understood than its terrible character seems to demand. Dr. Fox says that leprosy is not absolutely and always incurable, and suggests that a wealthy man who would endow a bed in the Skin and Cancer Hospital, so that lepers would come there, would be greatly facilitating the study of this disease. Nearly all the patients at Tracadie are descendants of two sisters, who about 100 years ago are said to have contracted the disease by washing clothes for sailors. In the beginning of the century there were about 100 cases in the neighborhood of Tracadie. A hospital was built on Sheldrake Island, not far from Tracadie, especially for lepers. Dr. Fox found a woman 80 years old in the lazaretto, who came there as a leper when a child. She was discharged as cured thirty years ago, but subsequently returned with fresh symptoms of the disease ; but in the doctor's opinion she will die of old age rather than leprosy.

Many of the patients have the worst form of leprosy—tubercular leprosy or leontiasis—so called because large bunches form over the eyes, giving the patient a lion-like, brutal expression. It is frightful to be in a room surrounded by such lepers. The macular lepers merely have bronze patches over the body. The disease is hereditary, but not contagious except by inoculation. To illustrate this phase of the disease the doctor said :

"A priest who visited the lazaretto caught the disease ; but I heard from Babineau that this priest in a spirit of bravado would take a pipe from a leper's mouth and smoke it. So he caught the disease from inoculation."

Dr. Fox does not think the disease is infectious. If he is correct the story of the origin of it in Tracadie must be rejected, and the infection of the women by washing the clothing of diseased sailors be treated as a myth. But the most important portion of the doctor's revelations must be that he knows of "six cases of leprosy in this city," and believes that "cases of leprosy exist in the Chinese quarter of New York, housed with other people, and perhaps intermarrying."—[*Scientific American.*

